# Platelet and Erythrocyte Extravasation across Inflamed Corneal Venules Depend on CD18, Neutrophils, and Mast Cell Degranulation

**DOI:** 10.3390/ijms22147360

**Published:** 2021-07-08

**Authors:** Angie De La Cruz, Aubrey Hargrave, Sri Magadi, Justin A. Courson, Paul T. Landry, Wanyu Zhang, Fong W. Lam, Monica A. Bray, C. Wayne Smith, Alan R. Burns, Rolando E. Rumbaut

**Affiliations:** 1 College of Optometry, University of Houston, Houston, TX 77204, USA; asdelacruz@uh.edu (A.D.L.C.); aubreych@stanford.edu (A.H.); srimagadi@gmail.com (S.M.); jacourso@central.uh.edu (J.A.C.); ptlandry09@yahoo.com (P.T.L.); wanyuzhang1218@yahoo.com (W.Z.); arburns2@central.uh.edu (A.R.B.); 2 Children’s Nutrition Center, Baylor College of Medicine, Houston, TX 77030, USA; flam@bcm.edu (F.W.L.); bray.monica@gmail.com (M.A.B.); cwsmith@bcm.edu (C.W.S.); 3 Michael E. DeBakey Veterans Affairs Medical Center, Center for Translational Research on Inflammatory Diseases (CTRID), Houston, TX 77030, USA

**Keywords:** inflammation, platelets, extravasation

## Abstract

Platelet extravasation during inflammation is under-appreciated. In wild-type (WT) mice, a central corneal epithelial abrasion initiates neutrophil (PMN) and platelet extravasation from peripheral limbal venules. The same injury in mice expressing low levels of the β_2_-integrin, CD18 (CD18_hypo_ mice) shows reduced platelet extravasation with PMN extravasation apparently unaffected. To better define the role of CD18 on platelet extravasation, we focused on two relevant cell types expressing CD18: PMNs and mast cells. Following corneal abrasion in WT mice, we observed not only extravasated PMNs and platelets but also extravasated erythrocytes (RBCs). Ultrastructural observations of engorged limbal venules showed platelets and RBCs passing through endothelial pores. In contrast, injured CD18_hypo_ mice showed significantly less venule engorgement and markedly reduced platelet and RBC extravasation; mast cell degranulation was also reduced compared to WT mice. Corneal abrasion in mast cell-deficient (Kit^W-sh/W-sh^) mice showed less venule engorgement, delayed PMN extravasation, reduced platelet and RBC extravasation and delayed wound healing compared to WT mice. Finally, antibody-induced depletion of circulating PMNs prior to corneal abrasion reduced mast cell degranulation, venule engorgement, and extravasation of PMNs, platelets, and RBCs. In summary, in the injured cornea, platelet and RBC extravasation depends on CD18, PMNs, and mast cell degranulation.

## 1. Introduction

Platelet recruitment to post-capillary venules at sites of acute inflammation has been demonstrated in a broad range of experimental models, often in association with neutrophil (PMN)-endothelial interactions [1,2,3,4,5]. Our studies in a mouse model of corneal epithelial abrasion demonstrate that an acute inflammatory response is required for efficient wound healing [4,6,7,8]. This model involves the recruitment of PMNs and platelets to the microvasculature (limbus) surrounding the cornea in an interdependent manner since systemic depletion of either cell type inhibits the other from being recruited [4].

Platelet extravasation at sites of inflammation is under-recognized and poorly understood, having been documented only in a relatively small number of studies [1,9,10,11]. Traditionally, the role of platelets was deemed to be limited to hemostasis and thrombosis; however, it is now evident that platelets contribute as essential mediators of inflammation in a broad range of conditions [12,13]. In our model of corneal epithelial abrasion, platelets and PMNs are essential mediators of effective wound healing responses, including wound closure and the promotion of nerve, keratocyte, and epithelial regeneration [4,6,14,15,16]. In this model, we found the extravasation of PMNs and platelets occurred across limbal blood vessels surrounding the avascular cornea [6,17]. While PMN transendothelial migration has been studied and reviewed extensively [18,19,20], the mechanisms responsible for platelet extravasation in inflammation are less clear.

Our prior study showed hypomorphic mutant mice expressing low levels of the leukocyte CD18 integrin (CD18_hypo_) have a marked reduction in total recruitment of platelets at the limbus (i.e., the sum of intravascular and extravascular platelets) following corneal epithelial abrasion with no apparent effect on PMN extravasation [15]. In the present study, we sought to define the role of CD18 on platelet extravasation in this model of inflammation, focusing on two relevant cell types that express CD18: PMNs and mast cells.

## 2. Results

### 2.1. CD18 Is Important for Platelet and Erythrocyte (RBC) Extravasation across Inflamed Venules in the Abraded Cornea

**Figure 1:**
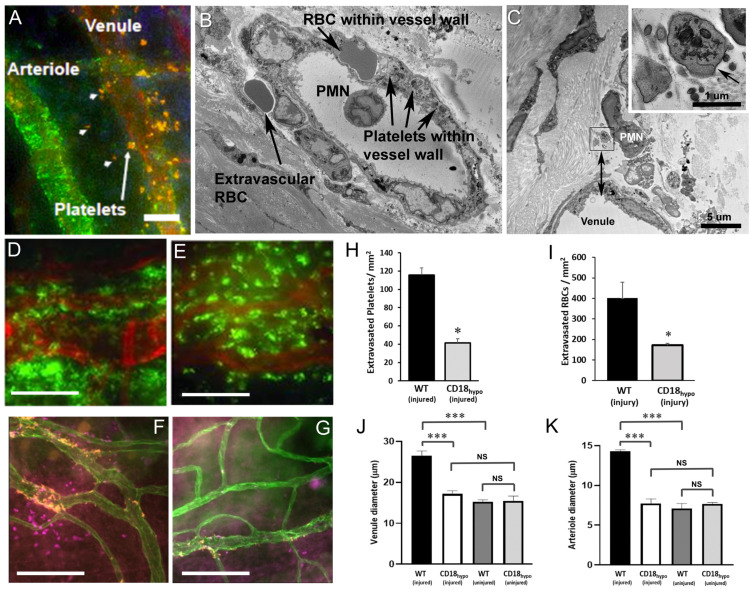
Blood cell extravasation after corneal abrasion in wild-type (WT) and CD18 hypomorphic (CD18_hypo_) mutant mice. Images of abraded corneas of WT (**A**–**E**) and CD18_hypo_ mice (**F**–**G**) at 8 h after epithelial abrasion. (**A**) Immunostaining (orange, anti-CD41) showing platelet extravasation from limbal vessels (arrowheads, extravascular platelets; arrow, platelets within the venule wall). A nearby arteriole identified by positive labeling for smooth muscle alpha-actin (green) showed no evidence of platelet extravasation. (**B**) Electron micrograph of an inflamed limbal venule showing an red blood cell (RBC) and several platelets beneath the endothelium but still within the venule wall. One extravascular RBC is located just outside the wall. (**C**) Electron micrograph showing an extravascular neutrophil (PMN) and several extravascular platelets next to a venule. The extravascular platelets are within the inset box immediately to the left of the PMN and the lower edge of the inset box is positioned 6 µm from the luminal endothelial surface of the venule (see double arrowhead). An enlarged view of the inset (upper right) shows an extravascular platelet positively identified by a surface-connected canaliculus (arrow). A second extravascular platelet (identity confirmed by serial sections not shown) is located to the lower left. (**D**,**E**) Immunofluorescence images from a WT mouse (**D**) and a CD18_hypo_ mouse (**E**) with large numbers of extravascular PMNs (green, anti-Ly6G) adjacent to limbal vessels (red, anti-CD31). (**F**,**G**) Immunofluorescence images show extravascular platelets (orange, anti-CD41) and extravascular RBCs (magenta, anti-TER119) adjacent to limbal vessels (green, anti-CD31) in a WT mouse (**F**) but absent from the CD18_hypo_ mouse (**G**). (**H**) Data showing extravascular platelet and (**I**) RBC counts at 24 h post-injury along with diameters of limbal venules (**J**) and arterioles (**K**) before and 24 h after epithelial abrasion (*n* = 6 mice per group, NS = Not Significant, * *p* ≤ 0.05 and *** *p* ≤ 0.001). Unlabeled scale bars = 15 µm (**A**); 10 µm (**B**); 50 µm (**D**,**E**); 100 µm (**F**,**G**).

In this first set of experiments, we sought to define the role of CD18 integrin expression on several key parameters of corneal inflammation. In wild-type (WT) mice, during the first 24 h post-corneal injury, immunofluorescence microscopy showed that platelets extravasate from limbal venules, not arterioles (Figure 1A). Examination of the inflamed venules by electron microscopy revealed an accumulation of not only platelets within the venule wall, but also red blood cells (RBCs, Figure 1B). Beyond the venule wall, we noted extravascular RBCs (Figure 1B) and platelets (Figure 1C) in the extracellular matrix where further observation showed platelets and RBCs remained at the limbus (in the peripheral cornea), in contrast to our earlier studies showing that PMNs leave the limbus and travel to the site of injury (center of cornea [15]). In a previous study, we reported that mutant CD18_hypo_ mice had normal levels of PMN extravasation but reduced total platelet recruitment (intravascular and extravascular) at the limbus, 24 h after corneal abrasion [15]. We now confirm those findings by demonstrating that extravascular platelet recruitment is reduced in CD18_hypo_ mice and add that RBC extravasation was similarly reduced (Figure 1D–I). Venule engorgement and arteriolar dilation following corneal abrasion were significantly blunted in CD18_hypo_ mice (Figure 1J,K). The microvascular diameter changes are consistent with a diminished inflammatory response in CD18_hypo_ mice [21]. Given isolated reports that human and mouse platelets express CD18 [22,23], we compared blood platelet counts and ex vivo platelet function under shear stress between CD18_hypo_ and WT mice. Platelet counts and platelet adhesion to collagen in a shear flow chamber were comparable between CD18_hypo_ and WT mice (Supplement, Figure S2). Similarly, platelet–PMN aggregates under resting conditions were comparable between CD18_hypo_ and WT mice (Supplement, Figure S3). Using flow cytometry, we were not able to detect evidence of CD18 expression on platelets derived from WT mice (data not shown).

### 2.2. PMNs Are Important for Platelet and RBC Extravasation across Inflamed Venules in the Abraded Cornea

**Figure 2:**
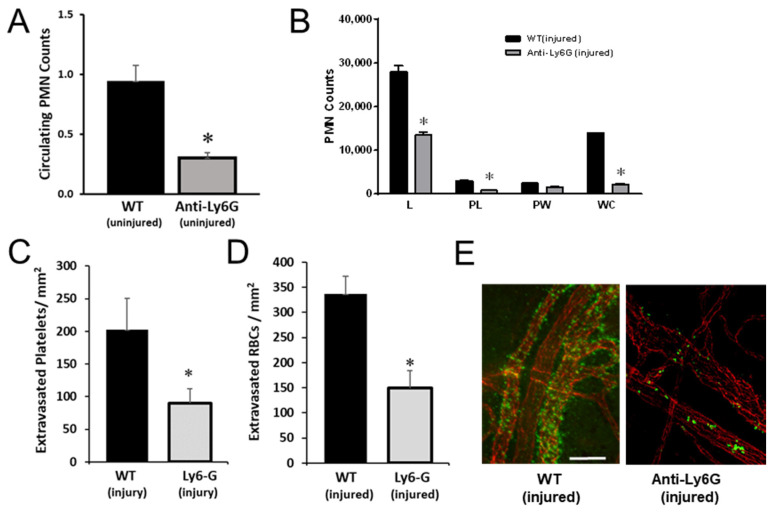
Anti-Ly6G antibody treatment reduces PMN extravasation and platelet and RBC recruitment. (**A**) Absolute circulating PMN counts were reduced in mice 24 h after control antibody WTor anti-Ly6G antibody injection. (**B**–**E**) Corneas were collected and immunostained 24 h after wounding. (**B**) PMN infiltration across the cornea (L = limbus, PL = paralimbus, PW = parawound, and WC = wound center). (**C**) Data on platelet extravasation at the limbus are plotted. (**D**) Data on extravascular RBCs at the limbus are plotted (**E**). Representative images of platelet (green, anti-CD41) extravasation from limbal venules (red, anti-CD31). *n* = 6 per group, * *p* ≤ 0.05 Bar = 40 µm.

Based on our prior studies showing interdependence between PMNs and platelet accumulation in corneal limbal vessels, we sought to determine whether PMNs mediated CD18-dependent platelet and RBC extravasation. To test whether PMNs are necessary for platelet extravasation, we undertook a set of experiments in which we used anti-Ly6G antibody to deplete circulating PMNs in WT mice just prior to corneal abrasion. As expected, this approach decreased the numbers of circulating PMNs by 60% (Figure 2A), similar to published reports from others using the same antibody clone [24,25]. Platelet counts (measured on blood samples obtained via cardiac puncture) were not statistically different: 400 ± 47 (isotype) vs. 531 ± 13 (anti-Ly6G), *n* = 3, *p* = 0.057. Twenty-four hours post-injury, mice treated with anti-Ly6G showed reduced PMN accumulation across the cornea (Figure 2B). In this setting, platelet (Figure 2C,E) and RBC (Figure 2D) extravasation were significantly reduced. These observations support the notion that PMNs are required for platelet and RBC extravasation across inflamed venules in this model.

### 2.3. Mast Cell Degranulation Is Critical for Platelet and RBC Extravasation in the Abraded Cornea

**Figure 3:**
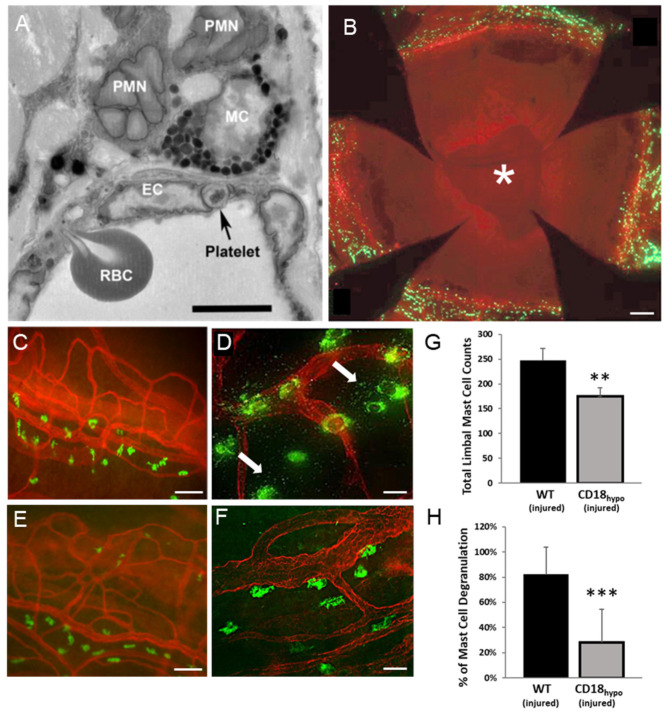
Reduced mast cell degranulation after corneal abrasion in CD18_hypo_ mice. (**A**) Electron micrograph of a WT mouse cornea 8 h after epithelial abrasion showing a platelet and an RBC in the process of traversing the inflamed endothelium of a limbal venule. Extravascular PMNs are in contact with a perivascular mast cell (MC). (**B**) Representative whole-mount image of a WT mouse cornea at 18 h after epithelial abrasion. Note the open wound (*). Perivascular mast cells (green, FITC-avidin) were detected at the limbus and not in the avascular cornea. (**C**–**F**) Immunofluorescence images of flat-mounted corneas where mast cells (FITC-avidin, green) are positioned next to the limbal venules (anti-CD31, red). (**C**) Uninjured WT cornea. (**D**) Twenty-four hours after an abrasion, mast cell degranulation (arrows) is apparent in the WT mouse. (**E**) Uninjured CD18_hypo_ cornea. (**F**) Twenty-four hours after an abrasion, mast cell degranulation is not evident (compare with WT image (**D**)). (**G**) Data showing limbal mast cell counts and (**H**) the percentage of mast cells undergoing degranulation (*n* = 6 per group, ** *p* ≤ 0.01 and *** *p* ≤ 0.001). Bar = 5 µm (**B**); Bars = 60 µm (**C**,**E**); Bars = 15 µm (**D**,**F**).

While our findings demonstrate a role for CD18 in platelet and RBC extravasation, a variety of leukocytes express CD18, including mast cells [26,27,28]. Electron microscopy of corneas following epithelial abrasion in WT mice has shown that PMNs, platelets, and RBCs extravasated from the limbal venules and PMNs came into contact with perivascular mast cells (Figure 3A). Since mast cells also express CD18, we performed additional experiments to determine whether mast cells mediated platelet and RBC extravasation. In WT mice, fluorescence labeling with FITC-avidin showed the limbal vasculature (labeled with anti-CD31 APC) surrounding the avascular cornea was replete with perivascular mast cells (Figure 3B). The mast cells were generally positioned adjacent to venules (Figure 3C), and in response to corneal abrasion, degranulation was evident (Figure 3D). While mast cell distribution was similar in CD18_hypo_ mice (Figure 3E), they showed little evidence of degranulation after wounding (Figure 3F). Quantitative analysis showed CD18_hypo_ mice had a slight, but significant, reduction (~20%) in perivascular mast cell numbers (Figure 3G) and a marked reduction (~4 fold) in mast cell degranulation after corneal abrasion (Figure 3H).

**Figure 4:**
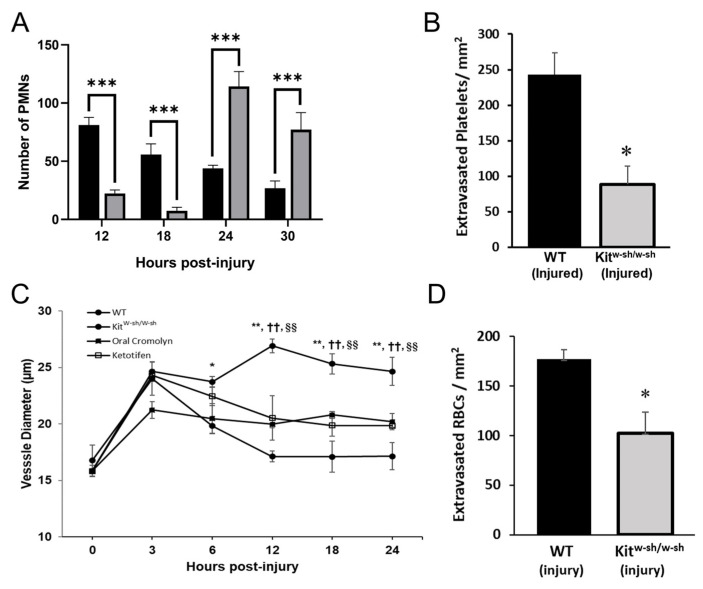
Inflammation after corneal abrasion in mast cell deficient (Kit^W-sh/W-sh^) mice and WT mice treated with mast cell stabilizers. (**A**) Numbers of extravasated PMNs at the center of the cornea were determined at different times after epithelial abrasion (black bars = WT mice; grey bars = Kit^W-sh/W-sh^ mice; *n* = 6 per group, *** *p* ≤ 0.001). (**B**) Numbers of extravasated platelets at the limbus 24 h post-injury were determined for WT and Kit^W-sh/W-sh^ mice. (*n* = 6 per group, ** *p* ≤ 0.01, *** *p* ≤ 0.001). (**C**) Venule diameters were determined at different times after corneal abrasion up to 24 h. (*n* = 6 per group, * *p* ≤ 0.05 and ** *p* ≤ 0.01, compared to Kit^w-sh/w-sh^; *n* = 6 per group, ^††^ *p* ≤ 0.01 compared to oral cromolyn; *n* = 6 per group, ^§§^ *p* ≤ 0.01 compared to ketotifen. (**D**) Numbers of extravasated RBCs at the limbus were determined at 18 h after corneal abrasion. (*n* = 6 per group, * *p* ≤ 0.05).

A previous study showed that WT and CD18_hypo_ mice had similar peaks for PMN extravasation following corneal abrasion, occurring between 12 and 24 h [15]; thus, we focused on this time frame. Following corneal abrasion, PMN recruitment to the wound center in mast cell-deficient (Kit^W-sh/W-sh^) mice was delayed at 12 and 18 h as evidenced by low numbers of infiltrating PMNs at the wound center compared to injured WT mice (Figure 4A). However, at 24 and 30 h post-abrasion, PMN recruitment increased and exceeded the levels found in WT mice (Figure 4A). Conversely, platelet extravasation in Kit^W-sh/W-sh^ mice was significantly depressed at 24 h post-abrasion (Figure 4B). Since mast cells play a critical role in arteriolar dilation and subsequent venular engorgement, it was not surprising to find attenuated venular engorgement in the mast cell-deficient Kit^W-sh/W-sh^ mice (Figure 4C); the mast cell deficiency was confirmed by an absence of mast cell-specific FITC-avidin staining (data not shown). Venule engorgement was reduced similarly in WT mice pre-treated with either of two mast cell stabilizers (cromolyn or ketotifen) (Figure 4C), supporting the idea that mast cell degranulation is required for venular expansion. Abraded corneas from Kit^W-sh/W-sh^ mice imaged at 24 h post-injury showed a significant reduction in RBC extravasation when compared to WT mice (Figure 4D).

### 2.4. Mast Cells Are Important for Recovery of the Epithelium in the Abraded Cornea

**Figure 5:**
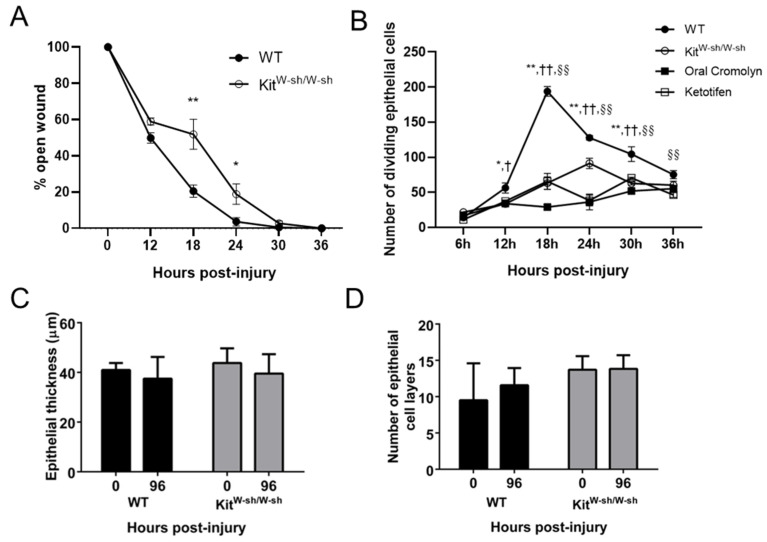
Changes in epithelial healing after corneal abrasion in Kit^W-sh/W-sh^ mice and WT mice treated with mast cell stabilizers. (**A**) The rate of epithelial wound closure was determined by fluorescein dye retention and expressed as a percent of the initial wound area. (*n* = 6 per group, * *p* ≤ 0.05, ** *p* ≤ 0.01). (**B**) Numbers of dividing basal epithelial cells were determined at different times after corneal abrasion. (*n* = 6 per group, * *p* ≤ 0.05, ** *p* ≤ 0.01 compared to Kit^W-sh/W-sh^; *n* = 6 per group, ^†^ *p* ≤ 0.05 and ^††^ *p* ≤ 0.01 compared to oral cromolyn; *n* = 6 per group, ^§§^ *p* ≤ 0.01 compared to ketotifen). (**C**) Epithelial thickness and (**D**) numbers of epithelial cell layers were determined from transverse histological sections of the cornea before injury (time 0) and 96 h post-abrasion.

In Kit^W-sh/W-sh^ mice, wound closure following corneal abrasion was delayed (Figure 5A). WT mice treated with mast cell stabilizers (cromolyn or ketotifen) prior to abrasion showed decreased basal epithelial cell division throughout the first 36 h post-injury and this decrease was mirrored in Kit^W-sh/W-sh^ mice (Figure 5B). Previous studies have shown that a reliable time for completion of epithelial thickness and restratification to be 96 h after injury [29,30]. By 96 h post-abrasion, the corneal epithelium was fully restored in each mouse strain as evidenced by the full restoration of epithelial thickness and number of epithelial cell layers to baseline levels (Figure 5C,D).

### 2.5. PMNs Are Important for Mast Cell Degranulation and Venule Engorgement

**Figure 6:**
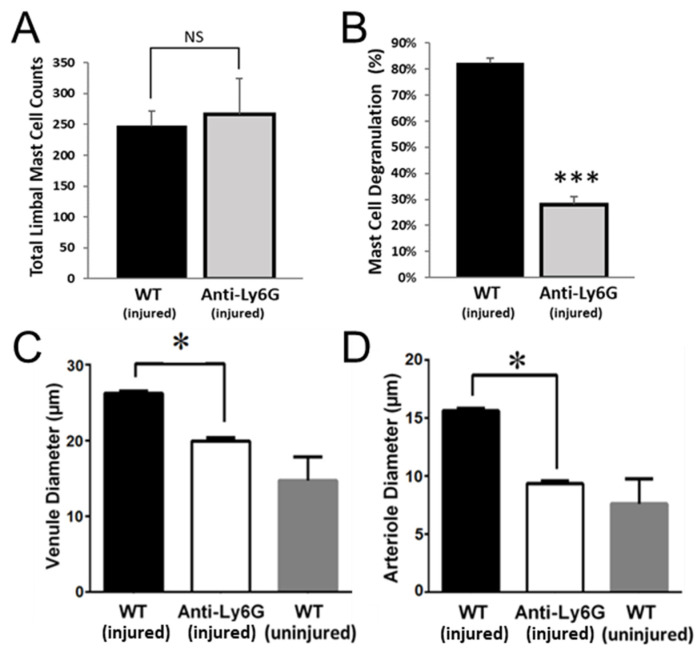
Anti-Ly6G antibody treatment reduces mast cell degranulation and limbal vessel expansion. (**A**–**D**) Corneas were collected and immunostained 24 h after wounding. (**A**) Analysis of limbal mast cell counts and (**B**) mast cell degranulation. (**C**) Venule and arteriole (**D**) diameters were measured at the limbus. *n* = 6 per group, * *p* ≤ 0.05, *** *p* ≤ 0.001 and NS = Not Significant.

PMN interactions with mast cells are thought to contribute to mast cell degranulation [31]. Indeed, as noted earlier, electron microscopy of abraded corneas from WT mice revealed extravasated PMNs in contact with mast cells (Figure 3A). To test whether PMNs are necessary for mast cell degranulation, we quantified mast cell number and degranulation in mice treated with anti-Ly6G antibody. In these mice, limbal mast cell numbers were unaffected (Figure 6A) but mast cell degranulation was largely prevented (Figure 6B), and limbal venule engorgement and arteriole dilation were significantly blunted (Figure 6C,D). These observations support the notion that mast cell degranulation requires PMN extravasation, and mast cell degranulation is required for platelet and RBC extravasation across inflamed venules in this model.

### 2.6. Platelet Extravasation across Inflamed Venules Is Accompanied by RBC Extravasation: Role of Disruption of Microvascular Wall Integrity

**Figure 7:**
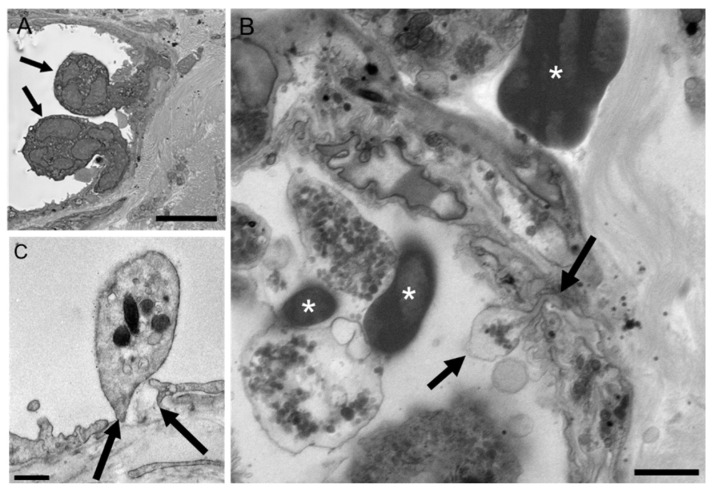
Representative electron micrographs of PMN and platelet extravasation in WT mice 8 h after corneal abrasion. (**A**) Two PMNs (arrows) engaged in diapedesis across the venular endothelium. (**B**) Extravasating platelet (bounded by two arrows) in the process of crossing the endothelium. Intravascular and extravascular RBCs (*) are also evident. (**C**) Intravascular platelet positioned over a discontinuity (arrows) in the endothelium. Small gold particles (5 nm) decorate the platelet surface and denote platelet-specific CD42b immunolabeling. Bar = 5 µm (**A**); Bar = 2 µm (**B**); Bar = 0.5 µm (**C**).

**Figure 8:**
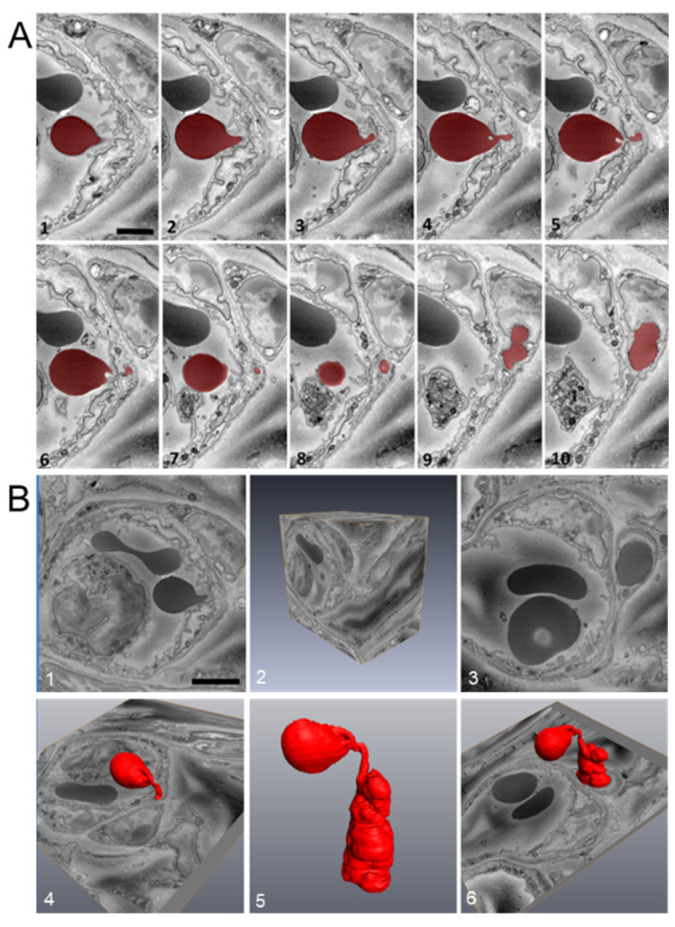
RBC extravasation after corneal abrasion. (**A**) Sequential images (1–10) were acquired using serial block-face scanning electron microscopy (SBF-SEM) and used to confirm RBC (colored red) passage across the venular endothelium. (**B**) Three-dimensional reconstruction analysis of the complete image stack (part of which is shown in panel (**A**)) illustrates the high degree of RBC deformability (panel B5) needed to pass through the endothelium.

In WT mice euthanized 8 h post-abrasion, ultrastructural observations of inflamed venules showed PMNs, platelets, and RBCs in various stages of extravasation (Figure 7 and Figure 8). For each cell type, passage across the endothelium involved close contact between the blood cell plasma membrane and the endothelial cell plasma membrane as the blood cell passed through the endothelial pore. The pore diameter for PMN passage ranged from 0.3 to 2 µm whereas platelets consistently passed through sub-micron pores (Figure 7B,C). RBCs also passed through sub-micron endothelial pores and exhibit a high-level of deformability during the process (Figure 8A,B). Like platelets, RBCs were observed migrating singly across the endothelium and in no particular relationship to adherent or transmigrating PMNs.

### 2.7. Extravasated RBCs and Platelets Are Phagocytosed by Perivascular Macrophages

**Figure 9:**
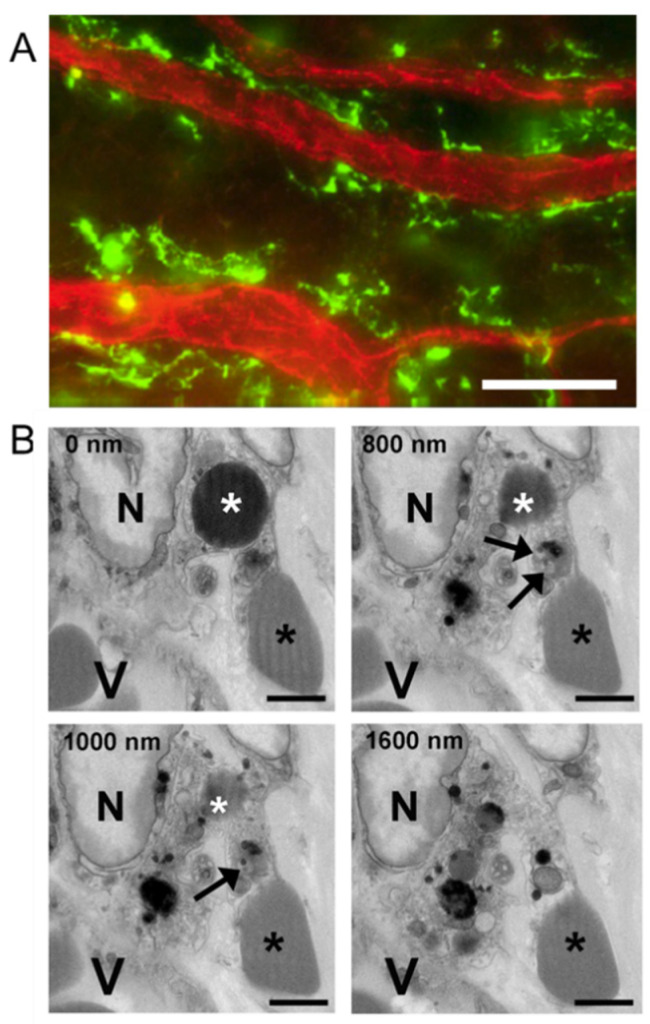
Perivascular macrophages and RBC and platelet phagocytosis after corneal abrasion. (**A**) Immunofluorescence image of perivascular macrophages (green, anti-CD301) lying next to limbal venules (red, anti-CD31). (**B**) Sequential SBF-SEM images reveal a perivascular macrophage containing a phagocytosed RBC and platelet. Top left in each panel indicates the depth of sectioning in nanometers. In each panel, a large macrophage nucleus (N) can be seen and the macrophage is located near a blood vessel (V). A phagocytosed RBC (white asterisk) is visible at 0, 800, and 1000 nm depths within the macrophage cytoplasm, but not at 1600 nm. A phagocytosed platelet containing dense granules (black arrows) is visible at 800 and 1000 nm depths but is absent at 0 and 1600 nm. An extravascular RBC (black asterisk) is in contact with the macrophage throughout the series. Bar = 25 µm (A); Bar = 2µm (B).

We have already reported on the fate and contribution to wound healing of PMNs and platelets [6]. Since macrophage phagocytosis of RBCs has been reported to influence the macrophage M1/M2 phenotype (i.e., pro- or anti-inflammatory) and thereby potentially influence the inflammatory response [32], we examined the injured cornea of WT mice for evidence of macrophage erythrophagocytosis. Immunofluorescence light microscopy showed limbal venules are associated with large numbers of perivascular macrophages (Figure 9A). Electron micrographs confirmed perivascular macrophages phagocytosed not only extravascular RBCs, but also extravascular platelets (Figure 9B).

## 3. Discussion

The purpose of this study was to better define the role of CD18 on platelet extravasation in a mouse model of central corneal epithelial abrasion, focusing on two relevant cell types that express CD18: PMNs and mast cells. Five novel findings are presented: (1) Following corneal abrasion, platelet extravasation at the limbus requires adequate levels of CD18 as evidenced by reduced numbers of extravasated platelets (but not PMNs) in mice with low CD18 expression (CD18_hypo_ mice). (2) This model of inflammation results in RBC extravasation, which parallels platelet extravasation. (3) Platelet and RBC extravasation are associated with venule engorgement and venule engorgement is markedly reduced when mast cell degranulation is absent or reduced. (4) Reductions in platelet and RBC extravasation are associated with delayed wound closure and diminished epithelial cell division. (5) Extravasated RBCs are readily phagocytosed by perivascular macrophages.

CD18 integrins are expressed on a variety of leukocytes, including mast cells [33]. On PMNs, CD18 integrins allow for the adhesive engagement of endothelial ICAM-1 and ICAM2 at sites of inflammation [34,35]. The CD18_hypo_ mutant mice express low levels of CD18, which is sufficient for PMN adhesion and emigration from the limbal venules after corneal abrasion, which is indistinguishable from that occurring in WT mice in terms of the timing of extravasation and the numbers of extravasated PMNs [15]. This is very different from what occurs in mice lacking CD18 expression (CD18 null), which shows a marked delay (24 h) in PMN extravasation following corneal abrasion [8].

In theory, CD18-dependent platelet adhesion to migrating PMNs is one possible mechanism for transporting platelets across the inflamed venular endothelium in WT mice. However, such an event was never observed in the current study despite obtaining electron micrographs of the inflamed limbus from more than 100 wounded corneal samples. Instead, platelets and RBCs appeared to cross the endothelium independently of PMNs and of one another, passing through sub-micron discontinuities or pores in the endothelium. We acknowledge that we cannot entirely exclude that platelet (or RBC) extravasation may depend in part on their direct adhesion to PMNs via CD18, and these adherent interactions may be decreased in CD18_hypo_ mice. CD18-dependent adhesion of leukocytes to platelets may occur via several binding partners on platelets, including glycoprotein 1bα [36,37], intercellular adhesion molecule 2 (ICAM-2, [38,39]), and glycoprotein IIb/IIIa (via fibrinogen, [40,41]). Although we found no differences in platelet-PMN aggregates under basal conditions, it is conceivable that CD18_hypo_ mice have reduced platelet-PMN aggregates following platelet activation since anti-CD18 antibodies have been shown to inhibit platelet-leukocyte aggregates induced by platelet activation [42]. In addition, CD18-dependent adhesion of leukocytes to RBCs has been reported via ICAM-4 on RBCs [43]. Further, direct adhesion of mast cells to platelets or RBCs would seem to be an unlikely initial mediator of platelet and RBC extravasation, given the perivascular localization of mast cells (Figure 3). To explain the CD18-dependent requirement for platelet (and RBC) translocation across the endothelium in the absence of direct adhesion, one could envision that CD18-dependent PMN adhesion to inflamed endothelial cells leads to focal PMN degranulation and loss of microvascular integrity with the formation of transient endothelial discontinuities or pores through which platelets and RBCs could pass. While directional migration of platelets (chemotaxis and haptotaxis) has been reported in some studies [11,44,45,46], this would not explain the associated extravasation of RBCs. Thus, a common driving force behind platelet and RBC translocation would more likely be the hydrostatic pressure gradient [47] which fits conceptually with our observation that extravasation is from engorged (larger diameter) venules where vascular pressures are elevated [48]. During platelet and RBC passage across the endothelium, the diameter of the endothelial pores can be very small (sub-micron), yet both cell types appear capable of squeezing through the small opening even though platelets possess a rigid microtubule-based cytoskeleton and are measurably stiffer (less deformable) than RBCs [49,50,51]. The idea that platelet and RBC extravasation is driven by hydrostatic pressure is consistent with our observations in both CD18_hypo_ mice and mast cell-deficient Kit^W-sh/W-sh^ mice where venule engorgement is markedly reduced compared to WT mice following corneal abrasion. The reduced engorgement would be consistent with a reduction in hydrostatic pressure, which in turn may be responsible for the observed reduction in platelet and RBC extravasation in these animals. Whether platelet chemotaxis contributes to their extravasation in this model of corneal abrasion remains to be determined.

The observation that venule engorgement is reduced in the injured corneas of CD18_hypo_ mice relates to the observation that mast cell degranulation was impaired. Mast cells release a broad variety of mediators capable of inducing vasodilation and, therefore, subsequently venular engorgement [52]. The impairment in CD18_hypo_ mice may relate to incomplete mast cell maturation due to reduced CD18 expression. Indeed, Rosenkranz and colleagues showed CD11b/CD18 expression is critical for mast cell homing and proper maturation within tissues [33]. However, there may be an alternative explanation. Our PMN depletion studies using anti-Ly-6G also showed markedly diminished mast cell degranulation and limited venule engorgement. A plausible explanation is that extravascular PMNs develop CD18-dependent adhesive interactions with mast cells leading to mast cell degranulation. This idea is supported by a previous study suggesting mast cell degranulation can occur through PMN adhesive interactions [31]. Indeed, we observed close contact between PMNs and mast cells (Figure 3A) and similar close contacts have been reported in other inflamed tissues (e.g., gut [53] and skin [54]). In vitro, adhesion between activated T cells and mast cells induces degranulation and is mediated, in part, by T cell CD11a/CD18 binding to mast cell ICAM-1 [55]. It is, therefore, reasonable to propose that PMNs could activate mast cells likewise. In the present study, the lack of mast cell activation in CD18 hypomorphic mice may be attributable to improper mast cell maturation compounded by insufficient PMN CD18 engagement with mast cell ICAM-1. Whereas the lack of mast cell degranulation in anti-Ly-6G-treated neutropenic mice would be attributable to fewer extravascular PMNs and, therefore, fewer PMN CD18 adhesive interactions with mast cells. Further, it is conceivable that the reduced mast cell degranulation in CD18 hypomorphic mice and in mice treated with anti-Ly-6G may be due to the reduction in platelet extravasation in these mice. Recent findings demonstrate that platelets are capable of promoting mast cell activation and degranulation in vitro and in vivo [56]. Clearly, additional studies are needed to test these hypotheses.

Crosstalk between RBCs and immune cells is another potential regulation point in inflammation, and in the abraded cornea this may occur during macrophage phagocytosis of extravasated RBCs. The macrophage can potentiate or attenuate the inflammatory response depending on its polarization state. The classic proinflammatory (M1) state can be triggered by phagocytosis of aged or damaged RBCs, whereas ingestion of younger undamaged RBCs tends to favor the anti-inflammatory (M2) state [32]. In the context of corneal inflammation caused by abrasion, the accumulation of PMNs at the limbus would provide a setting for increased reactive oxygen species (ROS) formation. In the presence of oxidative stress, extravasated RBCs would acquire an oxidized/senescent phenotype that, when phagocytosed by macrophages, could drive these cells toward the M1 state, thereby enhancing inflammation. In this scenario, the extravasated RBC would become more than just a bystander and it would take on a regulatory role in the inflammatory process. Similar to RBCs, extravasated platelets may play a regulatory role in the inflammatory process. Phagocytosis of platelets by macrophages (Figure 9) has been reported to promote the differentiation of monocytes into proinflammatory macrophages [57]. Further, extravasation of platelets is expected to result in platelet activation as a result of their exposure to collagen, a well-characterized platelet agonist [12]. Additional studies are needed to better understand the role played by extravasated RBCs and platelets in corneal inflammation and wound healing.

Our studies in the injured cornea provide evidence that mast cell degranulation at the limbus is not only essential for platelet and RBC extravasation, it is also essential for efficient wound recovery. We have shown previously that the time course for platelet and PMN recruitment is similar and the cells share an interdependence where depletion of one cell type reduces the recruitment of the other [4,15]. Platelets and PMNs are a rich source of VEGF, and we have shown that VEGF promotes acute epithelial wound closure and corneal reinnervation after abrasion [6]. Hence, the lack of platelet extravasation in the mast cell-deficient Kit^W-sh/W-sh^ mice, along with the delay in PMN extravasation, likely contribute to the observed delay in corneal epithelial wound closure during the first 24 h period post-injury. While the delay in PMN recruitment is overcome by 24–30 h post-injury in Kit^W-sh/W-sh^ mice (Figure 4A), no such recovery is seen in platelet recruitment (intravascular plus extravascular) even when the observation is extended to 36 h (Supplement, Figure S4). Hence, the opportunity for platelet recruitment seems limited and tied to PMN extravasation during the first 24 h post-injury.

Kit^W-sh/W-sh^ mutant mice used in our study, with an inversion upstream of the *Kit* gene, have a selective reduction of Kit expression and a well-characterized tissue mast cell deficiency [58]. Kit is also expressed by corneal epithelial cells. Using an in vitro corneal epithelial adhesion assay, Miyamoto and colleagues suggested that the binding of stem cell factor (SCF) ligand to the epithelial c-kit receptor positively influences epithelial cell attachment [59]. Indeed, the SCF/c-kit system is known to be important for epithelial cell maintenance [60]. For this reason, we included studies of WT mice where the SCF/c-kit system is intact but mast cell degranulation is blocked by the use of stabilizers (cromolyn or ketotifen). The data show wound healing is similar when comparing Kit^W-sh/W-sh^ and WT mice treated with mast cell stabilizers cromolyn or ketotifen; both show impaired wound healing. Consequently, the evidence is strong that the impaired wound closure in Kit^W-sh/W-sh^ mice is due to an absence of mast cells rather than a loss of epithelial c-kit/SCF binding.

Our previous studies indicate that an insufficient or delayed inflammatory response slows epithelial wound closure [4,6,30,61]. The delay in PMN extravasation and marked reduction in platelet extravasation in the Kit^W-sh/W-sh^ mice and WT mice treated with mast cell stabilizers is coincident with the absence of sustained venule engorgement. Larger, leaky blood vessels have been shown to allow for the passive, pressure-driven efflux of cells, a mechanism that could promote RBC and platelet extravasation following a corneal abrasion [62]. This observation is consistent with other studies demonstrating mast cells regulate blood vessel dilation separately from the autonomic dilatory response that accompanies injury [63]. Consequently, in our mast cell-deficient model of inflammation, sustained venule engorgement out to 24 h post-abrasion is not observed and suggests that mast cell degranulation is required. This finding is consistent with the notion that venule engorgement is dependent on mast cell degranulation and is associated with the efficient extravasation of PMNs, platelets, and RBCs. Although our studies demonstrate a very close association between venule engorgement and extravasation of PMNs, platelets, and RBCs, whether engorgement is necessary for extravasation of these cells remains to be determined. While reduced venular engorgement may contribute to the delay in PMN infiltration, another possibility in the Kit^W-sh/W-sh^ mice relates to the paucity of mast cell-derived chemotactic factors that normally promote PMN extravasation [5]. In either event, given the beneficial roles played by extravasated PMNs and platelets in corneal wound healing, it is not surprising then that any delay or reduction in their extravasation correlates with a delay in epithelial wound closure. This study broadens our understanding of the inflammatory responses following corneal wound injury, demonstrating a role for the extravasation of platelets and RBCs, as well as mast cell degranulation to the wound healing responses. As evident in our present and prior studies [4,6,14,15,16], the various cell types interact in a complex and interdependent manner, making it difficult to define precisely to what extent the physiologic functions of each individual cell type influences the course and resolution of the inflammatory response following corneal abrasion. Additional work seems warranted to define these complex cellular interactions.

In summary, the data suggest that in this model of corneal inflammation, platelet extravasation depends on CD18, mast cells and PMNs, with a central role for mast cell degranulation in the responses. Platelet extravasation is accompanied by RBC extravasation, with evidence of disruption of microvascular integrity. The role of platelet and RBC extravasation in microvascular inflammation remains to be fully defined.

## 4. Materials and Methods

### 4.1. Animals

Male and female C57BL/6J WT mice and Kit^W-sh/W-sh^ mice were 8–12 weeks old and purchased from Jackson Laboratories (Sacramento, CA, USA). The CD18_hypo_ mice were originally developed by Arthur Beaudet’s Laboratory at Baylor College of Medicine and have been backcrossed at least 10 generations with C57BL/6J mice [21]. All mice were housed and bred at the Baylor College of Medicine animal housing facilities.

### 4.2. Ethics Statement

All animals were handled according to the guidelines described in the Association for Research in Vision and Ophthalmology (ARVO) Statement for the Use of Animals in Vision and Ophthalmic Research and were reviewed and approved by the Baylor College of Medicine Institutional Animal Care and Use Committee policy guidelines (AN-2721, initially approved for these studies on 5 July 2014). Surgery was performed under pentobarbital anesthesia. Euthanasia was performed by isoflurane or pentobarbital overdose followed by cervical dislocation, or in some cases by exsanguination to obtain blood samples for blood cell counts. This was done either via inferior vena cava puncture or cardiac puncture, as indicated, under a surgical plane of anesthesia followed by bilateral thoracotomies.

### 4.3. Wound Protocol

Corneal wounding was performed as described previously [15,17]. Briefly, mice were anesthetized by administering pentobarbital (50 mg/kg body weight) by intraperitoneal (i.p.) injection. While viewing the corneas under a dissecting microscope, a 2.0 mm diameter trephine was used to demarcate the central epithelial region, and the epithelium of the demarcated area was removed using a golf-club spud. In some cases, mice received an i.p. injection of anti-Ly6G antibody (0.5 mg/mL in PBS, 0.25 mL per mouse) 24 h prior to wounding, thereby depleting the mice of circulating PMNs [6].

### 4.4. Treatment Protocol

To inhibit mast cell degranulation, WT mice received an oral administration of cromolyn (Sigma, 200 mg/kg by gavage, 12 h before injury) or a topical application of ketotifen (Bausch and Lomb, 2 × 5 μL onto to the ocular surface, 1 h before injury).

Complete blood counts were determined with an automated hematology analyzer (Siemens Advia 120, Siemens Medical Solutions, USA Inc. Malvern, PA, USA). For all mice, topical application of 0.1% fluorescein in sterile saline was used to evaluate the rate of epithelial wound closure. Excised corneal whole mounts were prepared for immunofluorescence microscopy and used to evaluate changes in limbal vessel diameters, numbers of extravasated platelets, PMNs, RBCs, and numbers of dividing epithelial cells [17].

### 4.5. Immunofluorescence Staining

Mice were euthanized as described above at specific intervals between 6 and 96 h after corneal injury. Excised corneas were fixed in phosphate-buffered saline (PBS, pH 7.2) containing 2% paraformaldehyde (Tousimus Research Corporation, Rockville, MD, USA) for 60 min at 4 °C, blocked for 30 min in PBS containing 2% BSA and permeabilized for 30 min with 0.1% Triton-X in PBS. Corneas were then incubated overnight at 4 °C with fluorescently-labeled antibodies (5–10 µg/mL) as follows: Platelets, PMNs, and blood vessels were labeled with anti CD41/PE (GP IIb), anti-Ly6G/FITC, and anti-CD31/ APC (or anti-CD31 FITC), respectively, (BD Bioscience, Pharmingen, San Jose, CA, USA). RBCs and mast cells were labeled with anti-TER-119 APC and FITC-Avidin, respectively (InVitrogen, ebioscience, Carlsband, CA, USA), while macrophages were labeled with anti-CD301 FITC (AbD Serotec, Kidlington, UK). To quantify degranulation of mast cells, we designated cells with evidence of extracellular staining for FITC-avidin as degranulated and those in which FITC-avidin remained intracellular were designated as not degranulated. Conjugated avidin is a well-characterized marker of mast cell granules [64]. Smooth muscle was stained with FITC conjugated α-smooth muscle actin (Sigma-Aldrich, St. Louis, MO, USA) to identify arterioles. DAPI (1 µg/mL; Sigma Aldrich, St. Louis, MO, USA) was used to stain cell nuclei. Labeled corneas received four equally spaced radial cuts so they could be flat-mounted in AIRVOL (Celanese, Dallas, TX, USA) and imaged using a DeltaVision wide-field deconvolution fluorescence microscope (GE Life Sciences, Pittsburg, PA, USA) with either an Olympus 20× dry lens or a 30× silicon oil lens.

### 4.6. Electron Microscopy

Corneas were processed for serial block-face scanning electron microscopy (SBF-SEM) as described previously in detail (24). Briefly, the corneas were fixed in 0.1 M sodium cacodylate buffer containing 2.5% glutaraldehyde, post-stained with heavy metals (Fe, OsO4, uranyl acetate, lead) before dehydration through an acetone series and embedding in Embed 812 resin (Electron Microscopy Sciences, Hatsfield, PA, USA) containing Ketjenblack EC600JD (Lion Specialty Chemicals Co., Tokyo, Japan). The resin-embedded blocks were sputter-coated with gold to reduce charging during block-face imaging. Tissue blocks were sectioned at 100 nm using a Gatan 3View2 system (Gatan, Pleasanton, CA, USA) mounted to a Mira 3 scanning electron microscope (Tescan, Pittsburgh, PA, USA). In some cases, platelets within the injured cornea were immunogold labeled and imaged using an FEI Tecnai 12 transmission electron microscope (ThermoFisher, Sugarland, TX, USA). These corneas were initially fixed with phosphate-buffered saline (PBS) containing 2% paraformaldehyde, permeabilized with 0.1% Triton-X 100 and sequentially labeled with a platelet-specific primary rat anti-CD42b antibody (10 µg/mL; InVitrogen, ebioscience, Carlsband, CA, USA) followed by a secondary goat-anti-rat IgG antibody conjugated to 5 nm gold particles (Nanoprobes, Yaphank, NY, USA).

### 4.7. Morphometric Analysis of Epithelial Thickness

The epithelial thickness of each cornea was measured using transverse sections (0.5 μm thick) from corneas prepared for electron microscopy stained with 1% toluidine blue O dye and examined by light microscopy with a 20× objective. The measurements were made from the top of the corneal epithelium to the basement membrane. Three central measurements (separated by 50 μm) were taken from each cornea and averaged.

### 4.8. Morphometric Analysis of Platelet and RBC Recruitment and Blood Vessel Diameter

For each flat-mounted corneal petal (four petals resulting from 4 radial cuts), a 9-panel montage of overlapping images was used to capture the full width (X), length (Y) and depth (Z) of the limbal vasculature using the DeltaVision microscope (GE Life Sciences, Pittsburg, PA, USA). Images were analyzed using Image j Software FIJI [65]. Dividing epithelial cells were counted from images acquired as digital image z-stacks (0.3 μm/slice) spanning the entire thickness of the cornea.

Maximum image projections were used to count platelet and RBC numbers which were then expressed relative to the limbal area as platelets/mm^2^ and RBC/mm^2^, respectively. The limbal area was determined by drawing a closed loop around the limbal vessels within each corneal petal. Previous reports show platelet recruitment to the site of inflammation enables adherent platelets to seal endothelial lesions (pores) from transmigrating PMNs [66]. To demonstrate platelet extravasation, we measured the average distance from the luminal surface of the endothelium to outer margin of the inflamed vessel wall (Supplement, Figure S1) and found the average wall thickness to be less than 5 µm. Based on these measurements, we limited our counts of extravasated platelets, to those platelets located outside the vessel wall at a distance of 5 µm or more away from the venule endothelium which was defined by immunofluorescent CD31 staining. The limbal venule endothelium was traced using imageJ software and a 5 µm line was drawn beyond the endothelium to account for the vessel wall thickness. Platelets located outside the 5 µm demarcated line were counted as extravasated. Platelets falling on or within the 5 µm demarcated line were excluded from the counts. The same maximum image projections and the same 5 µm distance from the venule endothelium were used to count extravascular RBCs and record blood vessel diameters (arteriole and venule).

### 4.9. Statistical Analysis

Data were analyzed using a Student’s *t*-test, a one-way or two-way analysis of variance (ANOVA) with Bonferroni’s multiple comparison post-hoc tests, as appropriate, using Prism software (GraphPad Prism version 8.0.0 for Windows, GraphPad Software, San Diego, CA, USA, www.graphpad.com, accessed on 7 July 2019). Data with unequal variances were log-transformed prior to analysis. A *p*-value ≤ 0.05 was considered statistically significant; data are reported as means ± SEM.

## Data Availability

The data presented in this study are available on request from the corresponding author.

## Supplementary Materials

The following are available online at https://www.mdpi.com/1422-0067/22/14/7360/s1.
