# Supplementary material for: Platelet and Erythrocyte Extravasation across Inflamed Corneal Venules Depend on CD18, Neutrophils, and Mast Cell Degranulation
Source: Int J Mol Sci. 2021 Jul 8;22(14):7360. doi: 10.3390/ijms22147360 (PMC8329926; doi:10.3390/ijms22147360)
Supplement: Supplementary file 1 [file ijms-22-07360-s001.zip › ijms-1250200-supplementary 2.pdf]

Supporting Information

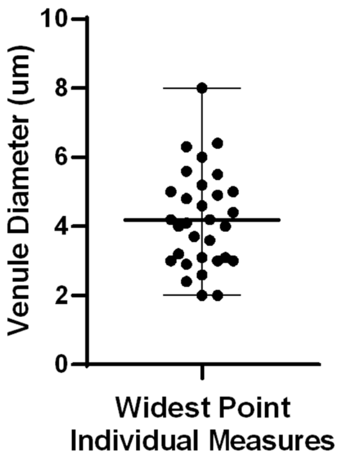

S1 Fig Caption. Venule diameters taken from 31 individual measures. Vessel diameters were taken at the widest point of 31 individual inflamed venules (μm).

S2 Text. Platelet adhesion to collagen under flow and platelet counts in CD18 hypomorphic mice ($CD18_{hypo}$) and WT mice.

Methods: Under isoflurane anesthesia, blood was collected via inferior vena cava venipuncture and anticoagulated with 80 μM PPACK (d-phenylalanyl-prolyl-arginine chloromethyl ketone). Blood platelet counts were measured with a Scil Vet ABC hematology analyzer (Scil animal care company). Flow chamber assays were conducted using a microfluidic BioFlux System (Fluxion Biosciences) with plates coated with type I collagen (25 μg/ml; Helena) and blocked with 5% Bovine serum albumin. Blood was labeled with 1 μM mepacrine and incubated at 37 °C for 20 min. Wild type and CD18 hypomorphic mice blood thrombus were captured simultaneously in the same optical field. The researcher performing the assay and image processing was masked

to mouse genotype. The labeled blood was perfused over the collagen-coated surfaces at two values of fixed shear stress (16 dyne/cm^2 and 30 dyne/cm^2). Thrombus formation was monitored with a Zeiss fluorescence microscope every 15 seconds, and analyzed using ImageJ at the end point of 90 seconds for statistical comparisons. Thrombus formation was calculated as the percent of coverage of the visualized channel (a constant surface area) above a set threshold of fluorescence.

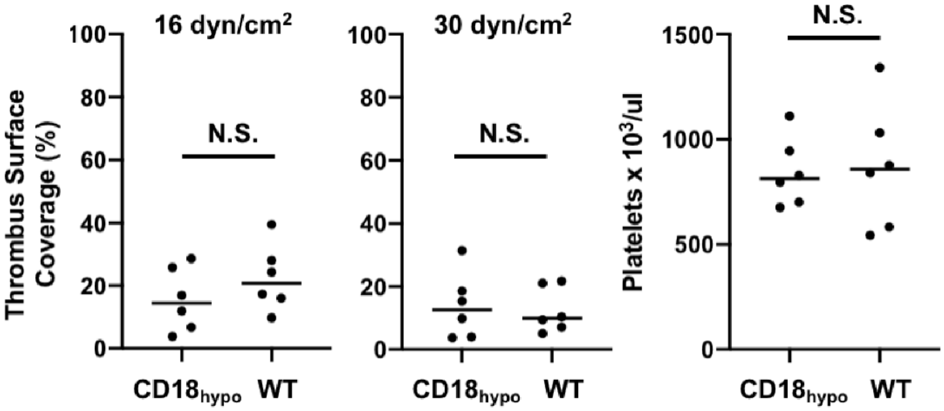

S2 Fig Caption. Platelet adhesion to collagen under flow and platelet counts in CD18 hypomorphic mice (CD18$_{hypo}$) and WT mice. Platelet adhesion under flow expressed as thrombus surface coverage, adhesion was comparable between CD18$_{hypo}$ and WT mice at a shear stress of either 16 dyn/cm^2 (left panel) or 30 dyn/cm^2 (middle panel). Blood platelet counts were comparable between CD18$_{hypo}$ and WT mice (right panel).

S3 Text. Platelet-neutrophil aggregates by flow cytometry in CD18 hypomorphic mice (CD18$_{hypo}$) and WT mice. Blood was collected as described in S2 and anticoagulated with 3.2% sodium-citrate; 0.5 µM prostacyclin was added to minimize platelet activation ex vivo. 100 µl of blood samples were incubated with fluorescein isothyocyanate-labeled anti-CD41 for platelets (0.2 µl of 0.5 mg/ml), APC-labeled anti Ly6G for neutrophils (1.0 µl of 0.2 mg/ml) and

phycoerythrin-labeled anti CD45 for leukocytes (0.5 µl of 0.2 mg/ml); all antibodies were obtained from BD Biosciences. After incubation for 30 minutes at room temperature, erythrocytes were lysed (1.0 ml of BD FACS Lysing Solution), samples were vortexed, incubated 15-30 min at room temperature (RT) protected from light, centrifuged at 300 x g at room temperature, washed with phosphate-buffered saline, fixed with 1% paraformaldehyde, and maintained at 4°C until analyzed with an imaging flow cytometer (Imagestream MKII, Millipore Sigma). Leukocytes were gated with the pan-leukocyte marker CD45 and within that gate neutrophils were detected with Ly6G; individually-labeled samples were used for color compensation. Platelet-neutrophil (PMN) aggregates were expressed as the percentage of Ly6G+CD45+ cells that also expressed CD41 (i.e., number of Ly6G+CD45+CD41+ Events / Total Ly6G+CD45+ Events, expressed as a percentage).

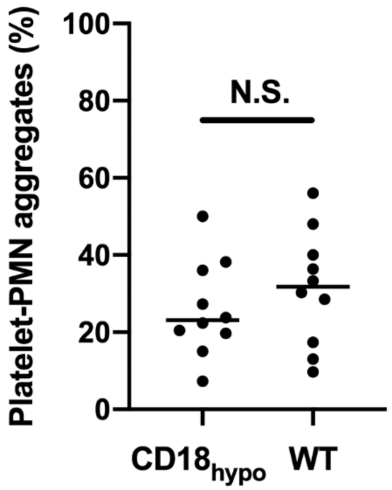

S3. Fig Caption. Platelet-neutrophil aggregates under basal conditions. The percentage of platelet-neutrophil (PMN) aggregates demonstrated a broad range under basal conditions but were comparable between $CD18_{hypo}$ and wild type (WT) mice. N=10 for each genotype

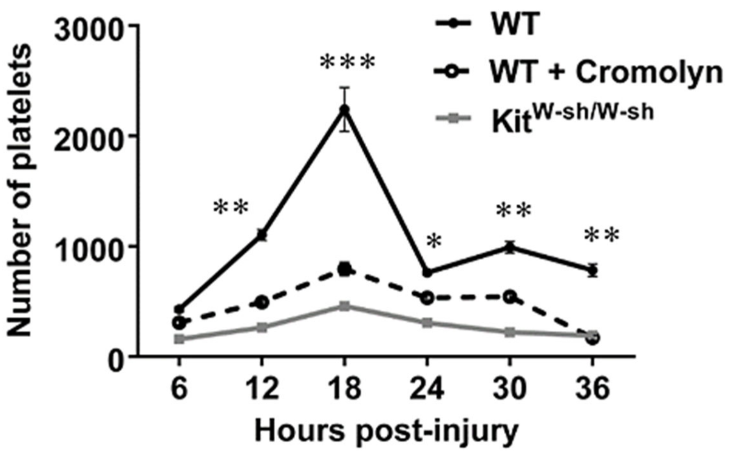

S4 Fig Caption. Platelet recruitment in WT mice, WT mice treated with cromolyn and Kit$^{w\text{-}sh/w\text{-}sh}$ mice. Platelet recruitment (intravascular plus extravascular) was determined at different times after corneal abrasion. (n=6 per group, * p ≤0.05 and ** p≤0.01 and *** p≤0.001compared to WT mice treated with cromolyn and Kit $^{w\text{-}sh/w\text{-}sh}$ mice).
